# *Arthrospira platensis* enriched with Cr(III), Mg(II), and Mn(II) ions improves insulin sensitivity and reduces systemic inflammation in equine metabolic affected horses

**DOI:** 10.3389/fendo.2024.1382844

**Published:** 2024-04-16

**Authors:** Artur Tomal, Jolanta Szłapka-Kosarzewska, Małgorzata Mironiuk, Izabela Michalak, Krzysztof Marycz

**Affiliations:** ^1^ International Institute of Translational Medicine, Wisznia Mała, Poland; ^2^ Department of Advanced Material Technologies, Faculty of Chemistry, Wrocław University of Science and Technology, Wrocław, Poland; ^3^ Department of Experimental Biology, Faculty of Biology and Animal Science, Wrocław University of Environmental and Life Sciences, Wrocław, Poland

**Keywords:** equine metabolic syndrome, insulin sensitivity, inflammation, feed additive, *Arthrospira platensis*, biosorption, metal ions

## Abstract

Equine metabolic syndrome (EMS) is a critical endocrine condition in horses, characterized by hyperinsulinemia, hyperlipidemia, and insulin resistance, posing a significant threat to their health. This study investigates the efficacy of supplementing EMS-affected horses with *Arthrospira platensis* enriched with Cr(III), Mg(II), and Mn(II) ions using biosorption process in improving insulin sensitivity and glucose tolerance, reducing inflammation, and mitigating obesity-related fat accumulation. Our results demonstrate that *Arthrospira* supplementation reduces baseline insulin and glucose levels, contributing to decreased adipose tissue inflammation. Furthermore, *Arthrospira* supplementation results in a decrease in body weight and improvements in overall body condition scores and cresty neck scores. Additionally, administration of *Arthrospira* leads to reduced levels of triglycerides and aspartate aminotransferase, indicating a decrease in hepatic adiposity and inflammation. These findings suggest that *Arthrospira*, enriched with essential micro- and macroelements, can be an advanced feed additive to enhance insulin sensitivity, promote weight reduction, and alleviate inflammatory processes, thereby improving the overall condition of horses affected by EMS. The use of *Arthrospira* as a feed additive has the potential to complement conventional management strategies for EMS.

## 1 Introduction

Microalgae, including diatoms, green algae, blue-green algae, and golden algae, offer diverse nutritional benefits and biological activities. *Arthrospira platensis*, a blue-green microalga, is particularly noteworthy for its recognition as a natural feed for horses due to its rich content of vitamins, minerals, carotenoids, proteins, and essential fatty acids (1, 2). The genus *Arthrospira*, formerly *Spirulina*, has demonstrated antimicrobial, antitumor, antiviral, anti-inflammatory, and hypocholesterolemia effects, along with radio-protective and metallo-protective properties that make it a promising superfood that may be considered a critically important ingredient in daily nutrition. The elevated protein and antioxidant content within *Arthrospira platensis* substantiates its pivotal role in metabolic homeostasis, providing indispensable amino acids requisite for protein synthesis, enzymatic catalysis, and overall cellular hemodynamics that are critically important for horses with metabolic disorders (3). The plethora of B-complex vitamins present in *Arthrospira platensis*, notably B12 and folate, intricately participates in metabolic pathways, thereby contributing substantively to energy metabolism, DNA synthesis, and amino acid metabolism (4, 5). Additionally, *Arthrospira*’s inclusion of essential fatty acids, particularly gamma-linolenic acid, assumes a noteworthy role in lipid metabolism, potentially impacting cardiovascular metabolic health (6). Furthermore, the bioactive constituents inherent to *Arthrospira platensis*, characterized by their antioxidant and anti-inflammatory attributes, confer an additional layer of metabolic modulation by safeguarding cellular entities against oxidative stress and orchestrating nuanced responses within the inflammatory milieu (1).

Equine metabolic syndrome (EMS) is a multifactorial endocrine disorder characterized by a cluster of metabolic abnormalities predisposing horses to several health complications, including laminitis (7). It is clinically characterized by insulin resistance, often followed by long periods of insulin dysregulation, culminating in impaired glucose utilization and the development of hyperinsulinemia (8, 9). The development of EMS is closely correlated with obesity; however, overweight *per se* is not required for EMS diagnosis, which makes it even more complicated for early diagnosis and prevention (7). Excessive adiposity, especially in the specific body part (regional fat deposits), is a hallmark of EMS. Adipose tissue acts as an endocrine organ, secreting adipokines that contribute to insulin resistance, a key component of EMS pathology (10). Our earlier studies demonstrated that adipose tissue accumulates excessive pro-inflammatory cytokines, including interleukin-6 (IL-6), in Welsh ponies with diagnosed EMS (11). The adiposity and/or obesity exacerbate insulin dysregulation and create a pro-inflammatory microenvironment, fostering systemic and regional inflammation (12). In addition to the aforementioned metabolic aberrations, EMS is intricately associated with features of systemic inflammation. Elevated levels of pro-inflammatory cytokines, such as tumor necrosis factor–alpha (TNF-α) and IL-6, have been earlier shown in horses with diagnosed EMS (11). These inflammatory mediators further prompt insulin resistance and perpetuate a harmful cycle, linking metabolic and inflammatory perturbations.

The intricate interplay between systemic inflammation, obesity, and liver deterioration in the pathogenesis of EMS involves complex molecular and metabolic mechanisms. Obesity, characterized by dysregulated adipose tissue expansion, serves as a primary driver for the release of adipokines, pro-inflammatory mediators originating from adipocytes. The ensuing systemic inflammation, marked by elevated cytokines such as TNF-α and IL-6, establishes a deleterious milieu that extends its influence to hepatic tissues (13). The liver, a central metabolic organ, becomes a focal point for the repercussions of systemic inflammation (14). Excessive lipid deposition within hepatocytes, characteristic of hepatic steatosis, ensues, heralding the progression of liver deterioration. Concurrently, the heightened pro-inflammatory environment contributes to the establishment of liver insulin resistance, disrupting the normal insulin signaling cascades within hepatocytes. This derangement in hepatic insulin sensitivity subsequently instigates impairments in glucose homeostasis (8, 15). The intricate orchestration of these events underscores the integral role of systemic inflammation and obesity in mediating liver dysfunction and insulin resistance, thereby unraveling the complex pathophysiology of glucose impairment in the context of EMS. This nuanced understanding is paramount for elucidating the holistic impact of EMS on equine metabolic health and holds implications for targeted therapeutic interventions.

Magnesium (Mg), manganese (Mn), and chromium (Cr) emerge as crucial elements intricately involved in the molecular modulation of vital physiological processes, notably obesity, insulin sensitivity, liver insulin resistance, and systemic inflammation (16). At the molecular level, magnesium is an essential cofactor for enzymes crucial in glucose metabolism, exerting regulatory influence on insulin action and overall glucose homeostasis (17, 18). Simultaneously, manganese, a trace element integral to carbohydrate metabolism, contributes to insulin signaling pathways and plays a pivotal role in insulin synthesis and secretion from pancreatic β cells (19–21). Chromium (III) has demonstrated its significance by facilitating insulin binding and enhancing insulin receptor phosphorylation, thus actively participating in the molecular events governing insulin sensitivity and glucose uptake (22, 23). The collective influence of magnesium, manganese, and chromium extends to adipocyte metabolism, impacting lipid storage, adipokine secretion, and broader adipose tissue functionality (24). Importantly, in hepatocytes, magnesium’s involvement in glycogen synthesis, manganese’s contribution to hepatic gluconeogenesis, and chromium’s modulation of hepatic insulin receptor signaling collectively influence liver insulin resistance, emphasizing the integral role of these elements in hepatic metabolic regulation. Noteworthy molecular interactions between magnesium, manganese, and chromium also manifest in systemic inflammation, where these elements contribute to the modulation of cytokine production, oxidative stress, and inflammatory signaling cascades. Their molecular roles in antioxidant defense mechanisms further underscore their significance in mitigating oxidative stress, a pivotal contributor to insulin resistance and inflammation.

In this study, we were interested in whether supplementation of *Arthrospira platensis* enriched with Cr(III), Mg(II), and Mn(II) ions improves insulin sensitivity and reduces systemic inflammation in equine metabolic affected horses. Conducted research included horses of both sexes classified into the control group of healthy animals (n = 6) and EMS group before supplementation (n = 9) that was also tested after admission of enriched with micro- and macroelements *Arthrospira platensis* (EMS group after supplementation n = 9). We have found that a 3-month supplementation period significantly reduces systemic inflammation and improves insulin sensitivity and liver-related enzyme activity.

## 2 Materials and methods

### 2.1 Ethical approval

All experimental procedures were approved by the II Local Ethics Committee of Wroclaw University of Environmental and Life Sciences (Chelmonskiego 38C, 51-630 Wroclaw, Poland; decision no. 84/2018).

### 2.2 Preparation of feed additive

*Arthrospira platensis*, a commercially available microalga, was acquired from Muhle Ebert Dielheim GmbH (Germany). This microalga was enriched with metal ions—Cr, Mg, and Mn—by biosorption in accordance with the methodology described by Michalak et al. (25). Inorganic salts MgSO_4_·7H_2_O, Cr(NO_3_)_3_·9H_2_O, and MnSO_4_·H_2_O, purchased from Avantor Performance Materials Poland S.A. (Gliwice, Poland), served as sources of these elements. The concentration of *Arthrospira platensis* in the solution was 10 g/L. Experimental conditions such as the initial concentration of metal ions in the solution of 200 mg/L, solution pH of 5 (correction with NaOH or HCl), process time of 1 h were established on the basis of previous studies (2). After the biosorption process, the biomass was separated from the solution and dried in a dryer. The elemental composition of natural and enriched microalgal biomass was determined using the ICP-OES (inductively coupled plasma–optical emission spectrometry) technique according to the methodology described by Michalak et al. (2). The analysis was performed in three replicates, and results are presented as a mean.

When preparing the diet for horses, the content of elements in the enriched *A. platensis* biomass and all nutrients in the feed were taken into account, following the recommendations of KER (Kentucky Equine Research). Each horse’s diet was proposed, covering the demand for the elements as mentioned earlier.

### 2.3 Horses

Age-matched (5–13 years; mean ± SD, 9.0 ± 2.2 years) donor horses of both sexes were classified into the control group of healthy animals (n = 6) and EMS group before supplementation (n = 9) that was also tested after admission of enriched with elements *Arthrospira platensis* (EMS group after supplementation n = 9). Detailed characteristic of the animals in this study is presented in **Table 1**. Assignation to the control or experimental group was performed on the basis of extensive interview with the animal keeper, measurement of body weight, estimation of body condition score (BCS) and cresty neck scoring (CNS) system, palpation and visual assessment of the hoof capsule, resting insulin levels, and X-ray examination, as described by Basinska et al. (11) During the experiment, animals spent the majority of the day outside on a sandy paddock but were excluded from pasture. One hour per day of moderate intensity exercise (longing) was provided. At the time of examination, the horses were clinically healthy, there were no ongoing generalized or local inflammatory conditions and no signs of laminitis, the mares were not pregnant, and the horses had not been subjected to intense work in the 48 h prior to the examination.

**Table 1:** General classification of horses into experimental group (EMS horses) and control group (healthy horses).

EMS horse number	Age	Sex	Breed	BCS	CNS
1	5	Female	Polish half-breed	7.5	3.5
2	9	Female	Polish half-breed	9	2.5
3	8	Male	Polish half-breed	8	4
4	5	Female	Polish half-breed	8	3
5	10	Male	Polish half-breed	7	3
6	10	Female	Polish half-breed	8	2.5
7	9	Male	Polish half-breed	8	2.5
8	10	Female	Polish half-breed	8	3.5
9	11	Male	Polish half-breed	8	2.5
Mean	8.5			7.95	3
SD	2.2			0.5	0.5
Healthy horses number (control)	Age	Sex	Breed	BCS	CNS
1	8	Female	Polish half-breed	5.5	1
2	10	Female	Polish half-breed	5	1
3	7	Female	Polish half-breed	5	1
4	13	Male	Polish half-breed	5	1
5	9	Female	Polish half-breed	6	1
6	11	Female	Polish half-breed	5	1
Mean	9.7			5.3	1
SD	2.2			0.4	0

### 2.4 Dietary protocol

The tested *Arthrospira platensis* (pelleted) as well as control feed (pelleted hay) were manufactured by Mühle Ebert Dielheim GmbH (MED, Dielheim, Germany). Horses classified in the experimental group (suffering for EMS as described in Section 2.3) received 20 g pelleted *Arthrospira platensis* per 100 kg of body mass according to KER specifications, once a day. Healthy horses (without known EMS’s syndromes as described in Section 2.3) assigned to the control reference group and horses in experimental group received the same amount of low starch feed (Brandon XL, Medvetico GmBH, Swiss). The daily dietary protocol included timothy grass hay (1.5% per 1 kg of body weight) and water *ad libitum* in the case of all groups involved in the study. Clinical examination together with body weight measurement of each individual was performed before and at the end of the 3-month dietary trial.

### 2.5 Sampling

#### 2.5.1 Preparing the horse for examination

The horse was prepared for the placement of a venous catheter. The insertion site was washed and disinfected, and the hair was shaved. The skin was re-disinfected, and, under sterile conditions, an external jugular vein cannula was inserted. The catheter was sutured to the skin for the duration of the study to reduce stress and discomfort for the animal during subsequent blood samples.

#### 2.5.2 Fasting blood collection

Blood was collected from the catheter into tubes with anticoagulant (EDTA morphological tube 3 × 1 mL) for morphological examination and for obtaining plasma for ACTH (adrenocortinotropic hormone) level determination, into a biochemical tube (serum biochemical examination tube with granules, 2 × 10 mL), and into a glucose determination tube (tube for glucose and lactate determination with sprayed K_2_EDTA and NaF, 2 × 2 mL). One of the morphological tubes was immediately centrifuged using a portable centrifuge (800D Centrifuge) at 3,000 rotations/min for 10 min, and the obtained plasma was immediately frozen and delivered to the laboratory in this state. The blood in the morphological tube and for glucose determination was kept under refrigerated conditions, approximately 4°C–10°C, until delivery to the laboratory. The biochemical tube, after blood coagulation at room temperature, approximately 30 min, was centrifuged (800D Centrifuge) at 3,000 rotations/min for 10 min obtain serum for biochemical determinations, including insulin level determination. The obtained serum was also kept under refrigerated conditions until delivery to the laboratory.

#### 2.5.3 Administration of glucose-fructose syrup

The amount of syrup was calculated on the basis of the body weight of the animal. The horse was previously weighed using a portable livestock scale (120 cm × 220 cm - Transport Animal Weighting Platform). The syrup dose was 15 mL/100 kg body weight. Corn syrup (Karo Light Corn Syrup) was used, and it was administered directly to the animal’s mouth using syringes in the appropriate amount.

#### 2.5.4 Subsequent blood collections from the horse

Subsequent blood collections were carried out in the following cycle, dependent on the time of sugar administration to the animal: 5 min, blood glucose level examination; 30 min, blood glucose level examination; 60 min, blood glucose and insulin level examination; 90 min, blood glucose and insulin level examination; 120 min, blood glucose level examination; and 150 min, blood glucose level examination. All blood collections were preceded by flushing the catheter with sterile saline solution and withdrawing a minimum of 5 mL of blood before collecting the intended material for analysis.

### 2.6 Isolation and propagation of equine adipose-derived stromal cells

Qualification of horses, tissue collection, adipose-derived stromal cell isolation and immunophenotyping were previously described Marycz et al. (26). Equine adipose tissue from tail head region was collected under septic condition according to previously described protocol (27). Obtained tissue biopsies were then washed with Hanks’ Balanced Salt Solution with the addition of 1% penicillin/streptomycin, cut using surgical scissors, and, finally, digested with collagenase type I solution (0.1 mg/mL) for 40 min at standard conditions (37°C, 5% CO_2_). After centrifugation (1,200 × *g*, 10 min), supernatant was discarded, and the cell pellet was suspended in full medium. The immunophenotype of equine ASCs was determined by fluorescence-activated cell sorting (FACS) analysis using fluorochrome-conjugated monoclonal antibodies against: CD44, CD45, CD90, and CD105 (data not shown). For primary equine stem cells, Dulbecco’s Modified Eagle’s Medium (DMEM) with Nutrient F-12 was applied, whereas secondary cell culture was maintained with DMEM containing 4,500 mg/L of glucose (both supplemented with 10% of FBS and 1% of antibiotic-antimycotic solution). Cells were cultured at standard conditions (37°C, 5% CO_2_).

### 2.7 ELISA tests

Using enzyme-linked immunosorbent assay (ELISA) tests, the amount of TNF-α (Mouse TNF-α Quantikine ELISA Kit, R&D Systems, Minneapolis, MN, USA), IL-4 (My Biosource, San Diego, CA, USA), IL-10b (Thermo Fisher Scientific, Carlsbad, CA, USA), and IL-1 (R&D Systems, Minneapolis, MN, USA) was evaluated. Procedures and calculations were carried out in accordance with the manufacturer’s instruction. The absorbance was measured with a 96-well microplate reader (Epoch, BioTek) at 450 nm.

### 2.8 Analysis of relative gene expression

For gene expression analysis, cells were homogenized with TRI Reagent^®^. Total RNA was isolated by phenol-chloroform extraction and ethanol precipitation (28). The concentration and purity of isolated RNA samples were analyzed using a spectrophotometer (Epoch BioTek^®^, Winooski, VT, USA) at a 260/280 nm wavelength. The RevertAid RT Kit was used for DNA-free RNA preparation and gDNA-free total RNA to cDNA transcription. Total RNA (150 ng) was used as a template for a single reaction. Procedures were carried out according to the manufacturer’s recommendations. Quantitative real-time polymerase chain reaction was performed using the SensiFast SYBR & Fluorescein Kit (Bioline, Cincinnati, OH, USA). All qRT-PCR reactions were carried out on 2.5 μL of cDNA in a 10-μL reaction volume using 500 nM of each specific primer. The CFX Connect™ Real-Time PCR Detection System (BioRad) was used with thermal cycling conditions specified in **Table 2**.

**Table 2:** Thermal cycling conditions used in RT-qPCR reactions.

Cycles	Step	Temperature	Time
1 cycle	Initial activation	95°C	120 s
35 cycles	Denaturation	95°C	15 s
	Annealing	Optimal primer annealing temperature	10 s
	Elongation	72°C	10 s
1 cycle	Final Elongation	72°C	120 s

To verify the specificity of the product, melting-curve analysis was performed. The mRNA levels were normalized in relation to the glyceraldehyde 3-phosphate dehydrogenase (GAPDH) as a housekeeping gene. The data were analyzed by the 2−ΔΔCt method. The sequences of all primers used in this work are listed in **Table 3**.

**Table 3:** The sequences of primers for every qRT-PCR experiment used in this work.

Gene	Forward primer 5′ to 3′	Reverse primer 5′ to 3′	Amplicon length	Accession no.
GAPDH	GATGCCCCAATGTTTGTGA	AAGCAGGGATGATGTTCTGG	250	NM_001163856.1
GLUT-4	CACCTTCTGTGGGGCATTGA	CGGGTTTTCAACAGATCGGC	146	NM_001081866.2
IGF-1	ATCAGCAGTCTTCCAACCCA	GAACTGAAGAGCGTCCACCA	86	NM_001082498.2
IL-1	TATGTGTGTGATGCAGCTGTG	ACTCAAATTCCACGTTGCCC	352	XM_014852743.1
IL-6	CGTCACTCCAGTTGCCTTCT	GCCAGTACCTCCTTGCTGTT	225	XM_014830631.1
TNF-alpha	AAGTGACAAGCCTGTAGCCC	GGTTGACCTTGGACGGGTAG	254	XM_014831605.1

### 2.9 Histochemistry and immunohistochemistry

Immunohistochemistry was performed as described previously by Basinska et al. (11). In brief, adipose tissue sample (2 g) was collected from the base of the mane from each horse under local anesthesia (2% Lignocainum). The histological material was preserved in 10% buffered formalin for 24 h. Five-micrometer-thick sections were obtained on Microm HM 340E microtome (Zeiss, Germany) and placed on histological slides. Samples were subsequently deparaffinized with xylene and ethanol (decreasing concentrations from 100% to 70%) and washed with distilled water. Slides were stained with hematoxylin (Shandon™, Thermo Scientific US) for 8 min, rinsed in running tap water for 10 min, and stained with eosin (Shandon™, Thermo Scientific US) for 5 min. Sections were dehydrated by washing with ethanol (increasing concentrations from 70% to 100%), followed by xylene and sealed with dibutylphthalate polystyrene xylene (DPX) mounting medium (AquaMed, Poland). Analysis was performed using light microscope (Axio Imager A1, Zeiss). The 3-μm-thick tissue sections were cut, dewaxed, and rehydrated. Immunoperoxidase cell labeling was performed using polyclonal antibodies against glucose transporter type 4 (GLUT-4), insulin-like growth factor 1 (IGF-1), IL-1, IL-6, and TNF-α. Heat-induced antigen retrieval was performed as follows: slides were incubated in the target retrieval solution (pH 9.0) (Dako, Denmark) for 20 min. Endogenous peroxidase activity was blocked with 3% hydrogen peroxide, and slides were washed with TBS (tris-buffered saline) for 5 min each. Tissue samples were labeled with antibody solutions. Primary antibodies were incubated for 20 min at 20°C. Detection was performed with EnVision™ Systems (Dako, Denmark). Sections were counterstained with Mayer’s hematoxylin for 1 min, dehydrated, and sealed. Analysis was carried out with optical microscope (Axio Imager A1, Zeiss Germany).

### 2.10 Statistical analysis

The obtained results were analyzed by multivariate analysis of variance (MANOVA) using GraphPad Software (Prism 8.20, San Diego, CA, USA), and differences between groups were determined using Tukey’ *post-hoc* test. Statistically significant results were marked with an asterisk, respectively, for < 0.05 (*), < 0.01 (**), and < 0.001 (***). Prior to ANOVA, normality analysis was assessed by Shapiro–Wilks test, and homogeneity of variances was determined using Levene’s test. Results are presented as the statistical mean ± SD from at least three independent experiments. The area under curve for time course of blood glucose concentration or blood insulin concentration after the standardized combined oral glucose-insulin test was calculated by using the Trapezoid rule as described by Sasaki et al. (29).

## 3 Results

The chemical composition of the microalga *Arthrospira platensis* used in the current study has been published previously (1, 2). Composition analysis included total phenolic content, fatty acids, including polyunsaturated fatty acids (PUFAs), free and protein-bound amino acids, phycocyanin, vitamin C and E (1), and micro- and macroelements (2).

### 3.1 Production of *Arthrospira*-based feed additive

Biosorption of Cr(III), Mn(II), and Mg(II) ions made it possible to maintain biomass enriched in elements that are crucial for horses diagnosed with metabolic syndrome. The content of these elements in *Arthrospira platensis* biomass before sorption was as follows: chromium, 1.87 mg/kg d.m.; manganese, 30.9 mg/kg d.m.; and magnesium, 2,301 mg/kg d.m. After the sorption process, the content of these elements in the biomass increased to 4,685 mg/kg d.m., 2,225 mg/kg d.m., and 2,484 mg/kg d.m., respectively (**Table 4**). The content of chromium in the biomass after biosorption increased by 2,505 times, manganese by 72 times, and magnesium by 8%. The weakest increase in the case of magnesium results from the mechanism of the sorption process, one of which is the ion exchange between metal ions bound on the surface of the biosorbent (most often Mg, Ca, K, and Na ions) and ions found in the aqueous solution (2, 25). The analysis of the elemental composition of natural *Arthrospira platensis* biomass showed that, among these elements, the highest content included alkali metals like potassium (14,384 mg/kg d.m.) and sodium (14,638 mg/kg d.m.) and then alkaline earth metals like magnesium (2,301 mg/kg d.m.) and calcium (1,967 mg/kg d.m.) (2).

**Table 4:** Multi-elemental composition of *Arthrospira platensis* before and after biosorption determined by ICP-OES (mg/kg d.m.).

Biosorption enrichment			
Element/wavelength (nm)	Before biosorption (mg/kg d.m.)	After biosorption (mg/kg d.m.)	Fold increase
Chromium (Cr)/267.716	1.87	**4,685**	2,505×
Manganese (Mn)/257.610	30.9	**2,225**	72×
Magnesium (Mg)/285.213	2301	**2,484**	1.08×

### 3.2 Insulin and glucose sensitivity improves after *Arthrospira platensis* administration

**Figure 1:**
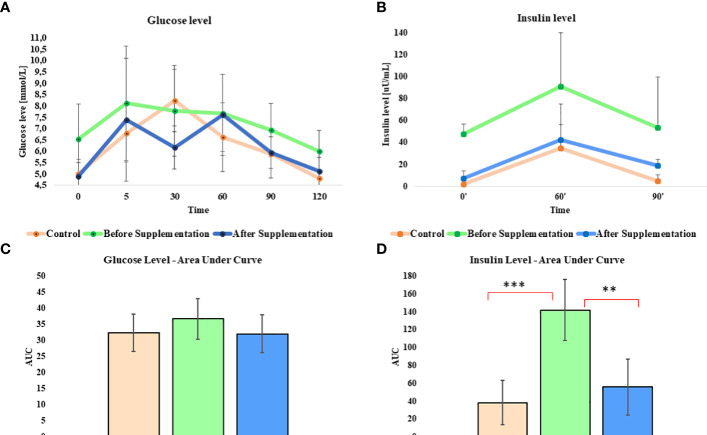
Glucose and insulin level before, during, and after the standardized combined glucose-insulin test. **(A)** Glucose and **(B)** insulin values measured per unit of time presented as a line chart. Mean area under curves for **(C)** glucose and **(D)** insulin during tests. Values are presented as a mean ± SD. Statistically significant differences are marked with an asterisk (**p < 0.01 and ***p < 0.001).

Oral sugar test was used to measure the influence of *Arthrospira platensis* supplementation on the level of glucose and insulin. Concentration of glucose obtained for EMS horses after supplementation in all measured time points was decreased in comparison to results before supplementation (**Table 5**; **Figure 1A**). Moreover, after supplementation, glucose level at the starting point of test (resting time 0’) decreased from 6.5 ± 1.5 mmol/L to 4.8± 0.7 mmol/L on average to comparable level with healthy horses’ group 5.0 ± 0.5 mmol/L. Glucose level of EMS horses after supplementation (5.1 mmol/L at 120’ time point) returns faster to resting value in comparison to glucose values before supplementation (6.0 mmol/L at 120’ time point), which reflects conditions observed in control group (4.8 mmol/L at 120’ time point). Although analysis of areas under the curve did not show any statistically significant differences between tested groups, the trend toward decreased glucose level between EMS and EMS supplemented with enriched *Arthrospira platensis* during oral sugar test was observed (**Figure 1C**).

**Table 5:** Measures of glucose and insulin responses before, during, and after the standardized combined glucose-insulin test.

Glucose level (mmol/L)						
Time (min)	Control	± SD	EMS before supplementation	± SD	EMS after supplementation	± SD
0’	5.01	0.49	6.53	1.55	4.89	0.75
5’	6.78	1.24	8.11	2.52	7.38	2.70
30’	8.25	1.37	7.78	2.00	6.18	0.95
60’	6.62	1.51	7.68	1.69	7.61	1.79
90’	5.88	1.05	6.94	1.17	5.94	0.70
120’	4.80	0.35	6.01	0.91	5.11	0.62
Insulin level (µU/mL)						
Time (min)	Control	± SD	EMS before supplementation	± SD	EMS after supplementation	± SD
0’	2.0	0.0	48.0	9.2	7.60	6.40
60’	34.9	21.5	91.2	49.1	42.7	32.6
90’	5.4	5.3	53.8	46.1	19.1	5.4

Values are presented as a mean ± SD.

Insulin level measured in EMS horses at resting conditions (time 0’) significantly (p < 0.001) decreased from 48.0 ± 9.2 µU/mL to 7.6 ± 6.4 µU/mL, before and after supplementation, respectively (**Figure 1B**). Peak of the insulin level reached after 60 min of glucose administration was nearly two-fold lower in EMS group after supplementation (42.8 µU/mL) in comparison to non-supplemented horses (91.2 µU/mL) and was comparable with control group (35.0 µU/mL). Supplementation results in overall improvement of insulin sensitivity in experimental group that reflects the return of insulin level after glucose administration to baseline resting values were comparable with those measured in control group. Measurement of areas under the curves confirmed significant decrease of insulin level in EMS treated with *Arthrospira platensis* enriched by biosorption in comparison to non-supplemented EMS horses (**Figure 1D**).

### 3.3 Reduction of body weight and general adiposity after *Arthrospira platensis* supplementation

**Figure 2:**
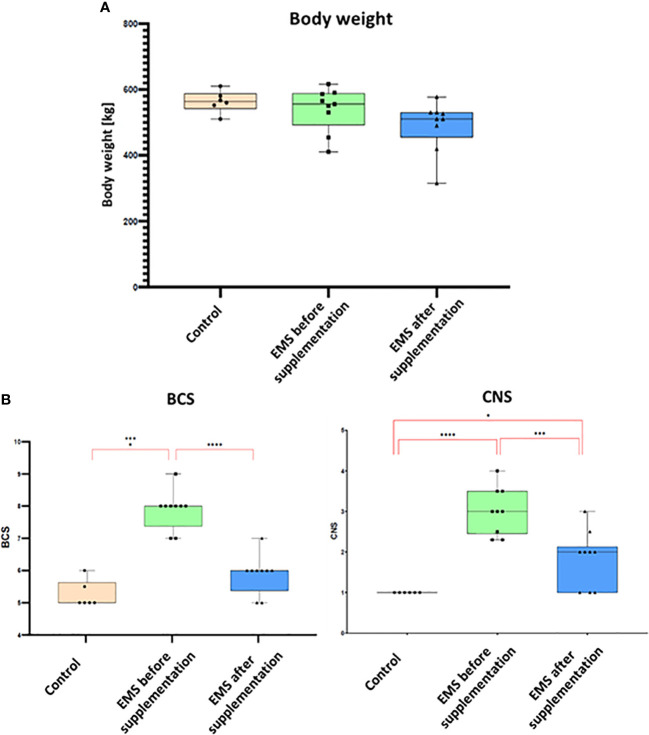
Body weight and adiposity parameters presented as bar graphs. **(A)** Individual body weights (kg) of EMS horses determined before and after supplementation. **(B)** Bar graph of BCS and CNS values. Results are expressed as mean ± SD. Statistically significant differences are marked with an asterisk (*p < 0.05, **p < 0.01, ***p < 0.001, and ****p < 0.0001).

To assess the influence of *Arthrospira platensis* supplementation on overall body condition of EMS horses, the following parameters including body weight, cresty neck score (CNS), and body condition score were examined before and after supplementation, and the results are presented in **Table 6**; **Figure 2**. Supplementation of *Arthrospira platensis* results in significant (p < 0.01) reduction of the body weight in all nine tested EMS horses from mean 560 ± 31 kg to 500 ± 49 kg mean in contrast to body weight of control horses 563 ± 33 kg). It corresponds to 12% and 67 kg on average weight reduction. No relationship between the loss of weight and sex was identified. By use of the nearest BCS integer value for obesity classification, all nine horses that were overweight or obese (BCS ≥ 7) before supplementation, improved their BCS (p < 0.01) after supplementation from average 7.85 ± 0.65 to 5.85 ± 0.65). After supplementation, BCS value for EMS horses (5.85) and healthy horses (5.25) were comparable. Supplementation results in substantial reduction of adipose tissue around the neck, which results in CNS decrease from 3.00 ± 0.50 to 1.75 ± 0.70 in the EMS group.

**Table 6:** Body weight and adiposity parameters in tested groups.

	Control	± SD	EMS before supplementation	± SD	EMS after supplementation	± SD
**Mean Body weight (kg)**	563	33	560	31	500	48
**Mean BCS**	5.25	0.40	7.85	0.65	5.85	0.65
**Mean CNS**	1.00	0	3.00	0.50	1.75	0.70

Mean values of body weight, body condition score (BCS), and cresty neck score (CNS) in control group and EMS horses before and after supplementation with *Arthrospira platensis*. Results are expressed as mean ± SD.

### 3.4 *Arthrospira platensis* supplementation positively affects inflammation response

**Figure 3:**
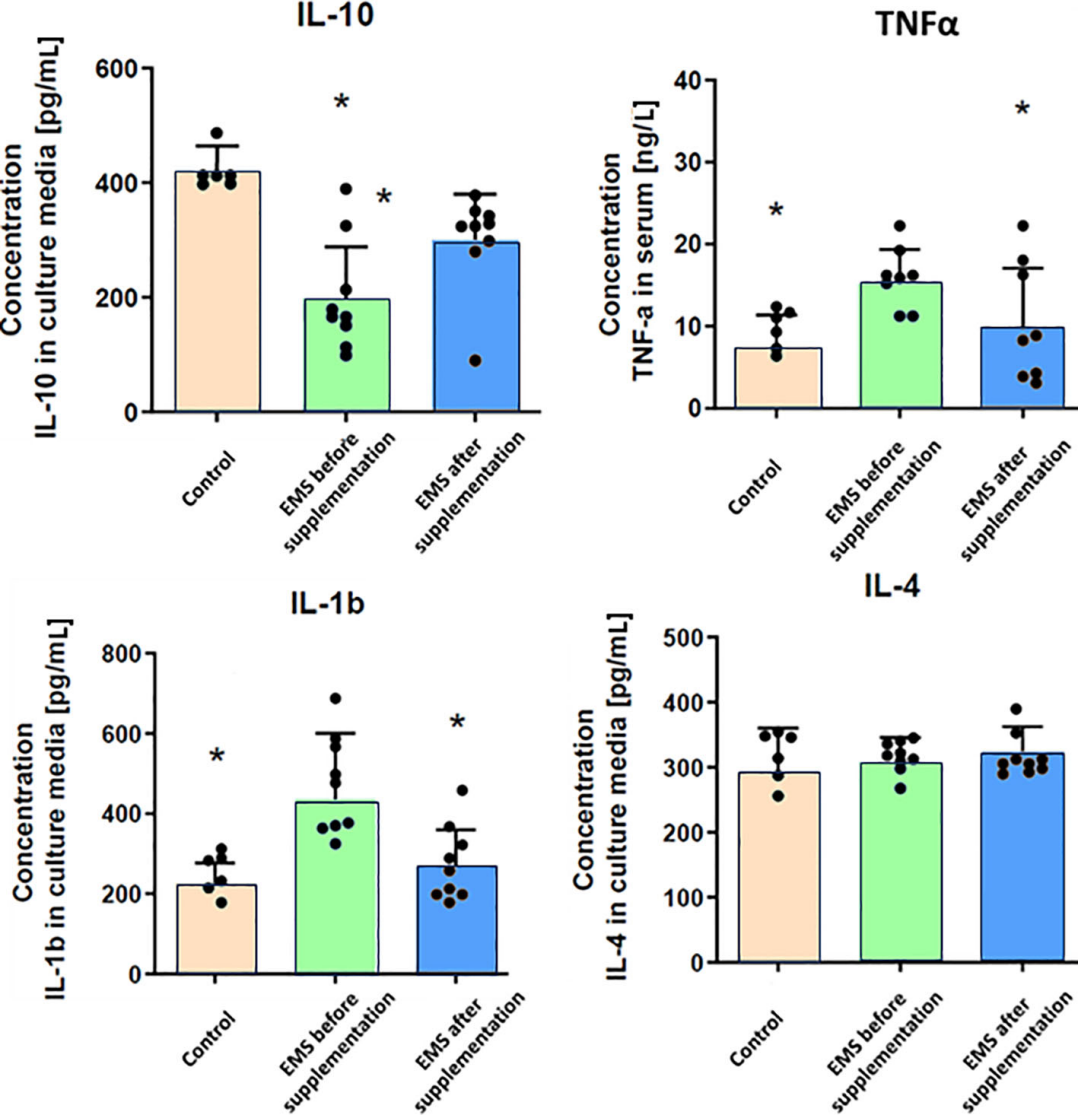
Inflammatory response-related enzymes in control group and EMS horses before and after supplementation. Results were obtained via ELISA assays and are presented as mean ± SD. Statistically significant differences are marked with an asterisk (*p < 0.05).

Through comprehensive analysis of interleukin levels before and after *Arthrospira platensis* supplementation, the intricate mechanisms underlying inflammation in horses were checked (**Figure 3**). Direct measurements of inflammatory factors through ELISA assays performed on blood collected from examined horses showed that, in group of EMS horses, the level of pro-inflammatory interleukin-1b (IL-1b) and TNF-α was significantly (p < 0.01) upregulated and the level of anti-inflammatory interleukin-10 (IL-10) was reduced in comparison to control group. Admission of bioactive *Arthrospira platensis* in supplementation resulted in reduced level of IL-1b and increased level of IL-10 and TNF-α, which almost equals the values measured in control group. Abundance of IL-4 did not change after supplementation and was comparable to all tested groups.

### 3.5 Liver-related parameters have been enhanced after *Arthrospira platensis* treatment

**Figure 4:**
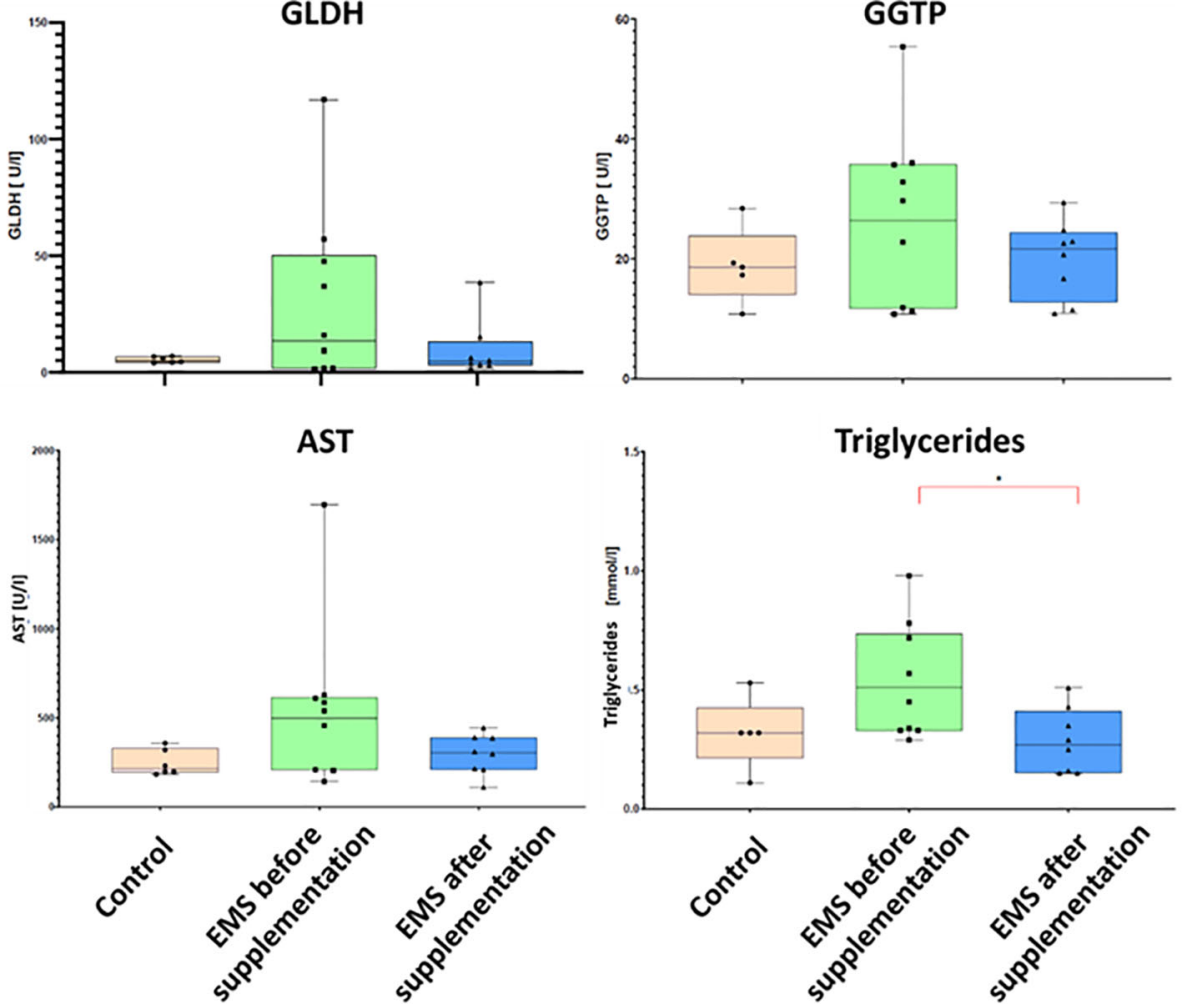
Liver metabolism parameters in control group and EMS horses before and after supplementation with *Arthrospira platensis* presented as bar graphs. Values of GLDH, GGTP, AST, and triglycerides are presented as mean ± SD. Statistically significant differences are marked with an asterisk (*p < 0.05).

To evaluate the impact of supplementation on liver metabolism, the level of ACTH, aspartate transaminase (AST), alkaline phosphatase (AP), glutamate dehydrogenase (GLDH), gamma-glutamyl transferase (GGTP), and triglycerides were checked (**Table 7**). Supplementation of bioactive *Arthrospira platensis* improved general condition of EMS horses’ livers according to measured parameters. EMS horses before supplementation have increased level of GLDH (29.9 U/L), GGTP (26.9 U/L), AST (438 U/L), and triglycerides (0.54 mmol/L) in comparison to both control group of healthy horses. After the supplementation, the level of aforementioned parameters decreased to values within the reference range (according to Rossdales Veterinary references): GLDH, 9.63 U/L; GGTP, 19.9 U/L; AST, 112 U/L; and triglycerides, 0.29 mmol/L (**Figure 4**). No significant changes were observed in the level of ACTH and AP after supplementation.

**Table 7:** Liver metabolism parameters in control group and EMS horses before and after supplementation with *Arthrospira platensis*.

Enzyme	Control	± SD	EMS before supplementation	± SD	EMS after supplementation	± SD
**ACTH (pmol/L)**	4.58	1.79	5.08	2.91	4.97	1.62
**AP (U/L)**	173.45	34.99	163.89	92.56	158.45	70.96
**AST (U/L)**	248.42	73.11	438.23	204.34	296.05	112.10
**GLDH (U/L)**	5.37	1.28	29.94	36.64	9.63	12.45
**GGTP (U/L)**	18.88	6.30	26.94	14.05	19.94	6.47
**Triglycerides (mmol/L)**	0.63	0.76	0.54	0.23	0.29	0.14

Values of ACTH, AP, AST, GLDH, GGTP, and triglycerides are presented as mean ± SD.

### 3.6 Bioactive *Arthrospira platensis* supplementation increases insulin sensitivity in adipose tissue of EMS horses

**Figure 5:**
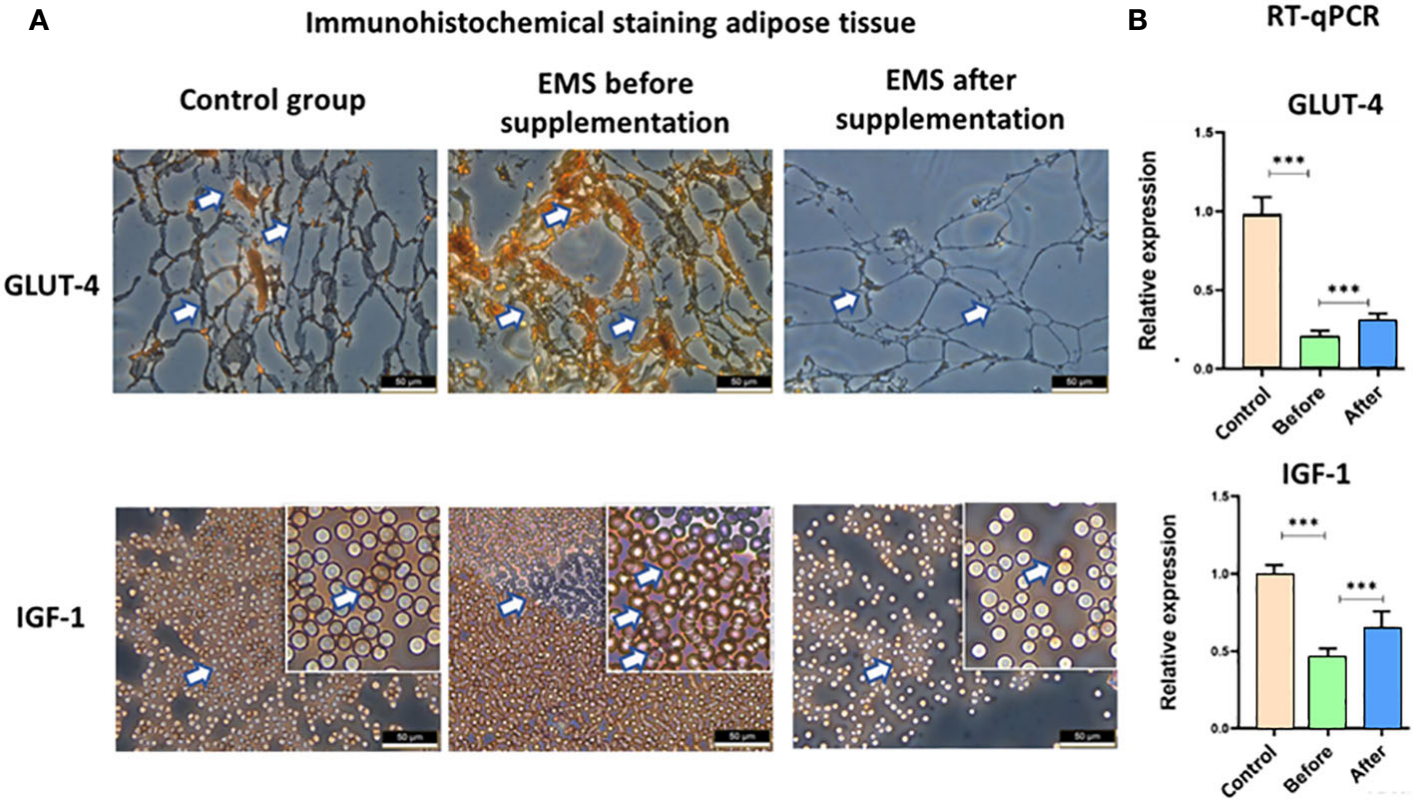
Examination of adipose tissue insulin sensitivity markers GLUT-4 and IGF-1. **(A)** Histology and immunohistochemistry examination of adipose tissue collected from healthy horses and equine metabolic syndrome horses before and after supplementation with *Arthrospira platensis*. ×40 magnification, scale bar = 60 µm. **(B)** Transcript level of GLUT-4 and IGF-1 measured in equine adipose-derived stromal cells. Values are presented as mean ± SD. Statistically significant differences are marked with an asterisk (***p < 0.001).

EMS is characterized by weaker insulin sensitivity observed in adipose tissue, which is connected with decreased level of GLUT-4 and IGF-1, which was also confirmed by immunohistochemical staining of adipose tissue. However, after *Arthrospira platensis* admission, we showed clear upregulation of these proteins in adipose tissue of supplemented EMS horses in comparison to EMS horses before supplementation (**Figure 5**). It was also confirmed in RT-qPCR analysis of transcripts levels, where significant increase of both genes’ mRNAs (p < 0.01) was observed after *Arthrospira platensis* admission to EMS horses; however, it was still not comparable with healthy horses.

### 3.7 Bioactive *Arthrospira platensis* supplementation reduces general inflammation in adipose tissue of EMS horses

**Figure 6:**
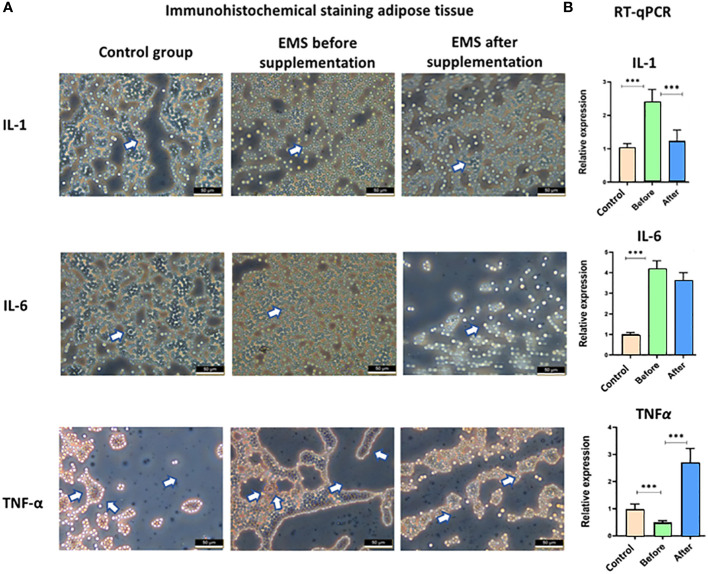
Examination of adipose tissue inflammation markers IL-1, IL-6, and TNF-α. **(A)** Histology and immunohistochemistry examination of adipose tissue collected from healthy horses and equine metabolic syndrome horses before and after supplementation with *Arthrospira platensis*. ×40 magnification, scale bar = 60 µm. **(B)** Transcript level of IL-1, IL-6, and TNFα measured in equine adipose-derived stromal cells. Values are presented as mean ± SD. Statistically significant differences are marked with an asterisk (***p < 0.001).

Moreover, the knowledge about inflammatory status was broadened by immunohistochemical staining of adipose tissue and transcript level evaluation of IL-1, IL-6, and TNF-α. Significant accumulation of these pro-inflammatory interleukins (p < 0.001) in EMS horses before supplementation in comparison to healthy horses was confirmed both at the protein level in immunostaining assay and at the transcript level by RT-qPCR analysis (**Figure 6**). After *Arthrospira platensis* admission, pro-inflammatory factors were clearly downregulated in EMS horses, which was observed by a weaker protein expression of IL-1, IL-6, and TNF-α in adipose tissues and by a lower transcript level of IL-1 and IL-6 in comparison to non-supplemented group. TNF-α transcript was upregulated after *Arthrospira platensis* supplementation in comparison to both healthy horses and horses before supplementation.

## 4 Discussion

EMS is a rapidly escalating, life-threatening endocrine disorder in horses. It is characterized by obesity, hyperinsulinemia, hyperlipidemia, insulin resistance, systemic inflammation, and oxidative stress (1, 30, 31). The primary contributors to the syndrome are high-calorie feeding strategies, insufficient exercise, and genetic predisposition (7, 10). Without prevention or treatment, EMS frequently leads to laminitis—a severely painful hoof condition that can necessitate euthanasia in affected horses. Consequently, there is an urgent need for effective treatments that target both nutritional and cellular factors, potentially including cellular therapies.

It is commonly known that only forage diet does not provide adequate concentration of protein, minerals, and vitamins for EMS horses. Thus, additional supplementation with commercially available feed balancer seems to be good solution. It has been demonstrated that oligosaccharide supplementation ameliorates insulin dysregulation (32), whereas a low dose of a synergistic polyphenol and amino acid blend including leucine improves metabolic health in EMS/insulin dysregulated horses (15). Furthermore, supplementation with omega-3 fatty acids would help to reduce problems associated with insulin resistance (33). Our own research has indicated that horses fed with a diet based on *Arthrospira platensis* supplementation lost weight and improved insulin sensitivity (1) and that *Arthrospira platensis* filtrates can considered as an agent with anticancer properties (34). Furthermore, it was showed that impairment of ASC isolated from EMS individuals (35) can be partially reversed by *Cladophora glomerata* enriched by biosorption process in Cr(III) extract (26). These examples showed the importance of proper supplementation with micro- and macroelements as well as oligosaccharides and polyphenols to prevent the EMS development. As a source of all mentioned compounds, algae additionally enriched with elements in the biosorption process, plays pivotal role in feeding of EMS horses.

*Arthrospira platensis* is widely recognized for its therapeutic benefits in conditions such as arthritis, cardiovascular diseases, and diabetes (36–38). Microalgal biomass enriched with microelement ions provides a highly bioavailable source of minerals to animals and can partially substitute the traditionally used inorganic salts as the primary source of microelements (39). This makes it particularly suitable for inclusion in the diet of horses diagnosed with EMS. Several research groups have proposed the supplementation of microalgae to horses suffering from EMS as a means to enhance glucose and insulin dynamics (40, 41).

In this study, we demonstrated that supplementation with *Arthrospira platensis* enriched with Cr(III), Mg(II), and Mn(II) ions increased insulin sensitivity in EMS-afflicted horses. Following oral sugar administration, the observed rise in both insulin and glucose levels was reduced after *Arthrospira* supplementation, although the response did not fully match the curves seen in healthy horses. These findings align with previous *in vivo* studies by Nawrocka et al. (1), which indicated that a diet incorporating *Arthrospira platensis* can enhance insulin sensitivity in horses. The observed clinical effect is attributed to the modulation of insulin signaling pathways as a result of improvement of proliferative activity and viability of ASCs affected by metabolic syndrome, as a result of their reduced senescence manifested by β-galactosidase activity, and as a result of reduced ASC apoptosis inducers involved in regulating cellular senescence progress and proliferation. Recent evidence suggests that even small, diet-compatible quantities of microalgal bioactive compounds can exert a significant regulatory effect on cellular signaling (42). Phenolic compounds in *Arthrospira platensis* are implicated in the regulation of redox signaling, mitigating the formation of reactive oxygen and nitrogen species (1).

Furthermore, our findings indicate that the incorporation of *Arthrospira platensis* into the daily diet of EMS-afflicted horses not only modulates insulin sensitivity but also prompts adipose tissue alterations, culminating in notable weight reduction. We have found that all horses that contribute to experimental group experience significant body weight reduction and decrease in fat along crest that results in overall improvement of body condition score. This beneficial effect aligns with previous reports (1, 37, 43, 44), wherein *Arthrospira* supplementation was associated with decreased fat deposition, particularly within the nuchal crest. Consequently, clinical parameters indicative of overall equine health, such as the CNS and body condition score, were significantly ameliorated post *Arthrospira* administration, approaching values observed in healthy equine cohorts.

Moreover, the high content of omega-3 fatty acids in *Arthrospira* (1), notably the long-chain polyunsaturated fatty acids such as eicosapentaenoic acid (EPA) and docosahexaenoic acid (DHA), is crucial for insulin regulation (45). DHA can directly modulate insulin signaling via the G-protein–coupled receptor 120, a known omega-3 fatty acid sensor essential for the insulin-sensitizing effects of these fatty acids. Binding of DHA to this receptor stimulates glucose uptake through the GLUT4 pathway, potentially elucidating the mechanisms underlying the results of our study. Additionally, EPA and DHA may indirectly influence insulin signaling by altering the composition of the cell membrane’s fatty acid profile. Altering the saturation level of DHA in cell membranes has been shown *in vitro* to enhance insulin-receptor binding and insulin action, emphasizing the importance of membrane lipid composition in cellular function (40, 46, 47).

Obesity in EMS is intricately linked to persistent inflammatory states (7), a connection underscored by our observation of reduced pro-inflammatory markers IL-1β and TNF-α alongside an elevation in the anti-inflammatory cytokine IL-10 within the serum. These results corroborate the findings from other research groups that have demonstrated the anti-inflammatory properties of *Arthrospira* (48–51). Such anti-inflammatory effects are attributed to the high concentrations of natural carotenoids, xanthophylls, and particularly phycocyanin, which contain the chromophore phycocyanobilin. As shown in a previous study, *Arthrospira platensis* contains 266 ± 23 mg of phycocyanin per 100 g of biomass (1). Phycocyanobilin has been shown to inhibit nicotinamide adenine dinucleotide phosphate oxidase activity, a pivotal contributor to oxidative stress within adipocytes and a modulator of adipokine and cytokine profiles that promote insulin resistance in hypertrophic adipose tissue (52). Therefore, *Arthrospira*’s capacity to mitigate oxidative stress in adipocytes may confer systemic anti-inflammatory and insulin-sensitizing benefits. It should be emphasized that this beneficial effect associated with decreased inflammation is observed not only for *Arthrospira* but also for several other extracts such as hydroalcoholic extract from *Proposis farcta* or Mulberry biomass in mice systems (53, 54). Moreover, the influence of *Arthrospira platensis* on YTHDF2 should be evaluated in the future perspectives as it was shown that it affects directly IL-6 inflammatory pathway by inhibiting high mobility group box 1 (HMGB1) release and janus kinase 2/signal transducer and activator of transcription 1 (JAK2/STAT1) signaling (55).

An examination of adipose tissue substantiated these systemic effects, with increased expression levels of GLUT-4 and IGF-1 mirroring the serological findings and reaffirming enhanced insulin sensitization. A concomitant decrease in pro-inflammatory cytokines IL-1 and IL-6 further validated the reduction in inflammatory response.

The pro-inflammatory milieu associated with the pathogenesis of EMS is characterized by an increased hepatic lipid accumulation. This condition is mediated by upregulated expression of TNF-α, suppressor of cytokine signaling 3, and Toll-like receptor 4, which are indicative of a heightened inflammatory response (56). In parallel, human metabolic syndrome exhibits a similar pathology, where ectopic fat deposition, particularly within hepatic tissues, precipitates local inflammation attributable to lipotoxicity, subsequently leading to liver fibrosis and hepatocyte apoptosis. In this context, our investigation assessed the beneficial effect of *Arthrospira*, biosorbent with magnesium, manganese, and chromium, on hepatic-related enzyme activity. The authors suggested that hepatic lipidosis caused the changes in liver metabolism. It is known that changes in liver metabolism could be exposed by variation in aspartate aminotransferase (AST) level, which functions to transaminate aspartate, which has been commonly used as a biomarker for hepatic injury (57). Therefore, the amount of AST in the blood is directly related to the extent of the liver tissue damage (58). Furthermore, liver damage is also manifested by increment of triglycerides, which normally are the main components of lipid in the liver and are derived from esterification of FFAs (59). In the setting of overnutrition and obesity, hepatic fatty acid metabolism is altered, commonly leading to the accumulation of triglycerides within hepatocytes. Post-supplementation analyses revealed a significant diminution in serum triglycerides and AST levels, which may be indicative of reduced hepatic adiposity and systemic inflammation.

The enriched *Arthrospira* has demonstrated efficacy as a sophisticated feed supplement, enhancing insulin sensitivity and dampening inflammatory processes, thereby potentially facilitating the overall condition of horses by diminishing adipose tissue proliferation. Furthermore, the incorporation of *Arthrospira* as a feed additive may offer synergistic benefits, reinforcing the standard management strategies employed in the treatment of metabolic syndrome.

## Data availability statement

The original contributions presented in the study are included in the article/supplementary material. Further inquiries can be directed to the corresponding author.

## Ethics statement

The animal studies were approved by II Local Ethics Committee of Wroclaw University of Environmental and Life Sciences (Chelmonskiego 38C, 51-630 Wroclaw, Poland; decision No. 84/2018). The studies were conducted in accordance with the local legislation and institutional requirements. Written informed consent was obtained from the owners for the participation of their animals in this study.

## Author contributions

AT: Data curation, Methodology, Writing – original draft, Writing – review & editing. JS: Data curation, Investigation, Writing – original draft. MM: Data curation, Investigation, Writing – original draft. IM: Data curation, Funding acquisition, Investigation, Methodology, Resources, Writing – original draft, Writing – review & editing. KM: Conceptualization, Formal analysis, Funding acquisition, Investigation, Project administration, Resources, Supervision, Writing – original draft, Writing – review & editing.
